# Supplementary material for: Anomalous universal conductance as a hallmark of non-locality in a Majorana-hosted superconducting island
Source: Nat Commun. 2022 Nov 5;13:6699. doi: 10.1038/s41467-022-34437-9 (PMC9637197; doi:10.1038/s41467-022-34437-9)
Supplement: Supplementary file 1 — Supplementary Information [file 41467_2022_34437_MOESM1_ESM.pdf]

Supplementary Information for "Anomalous universal conductance as a hallmark of non-locality in a Majorana-hosted superconducting island"

In this supplementary information, we will provide details concerning: (I) The effective low-energy Hamiltonian of the impurity and the conductance peak position; (II) The effective low-energy tunneling Hamiltonian, (III) Detailed derivation of the current following Eq. (5) of the main text, (IV) High temperature regime: detailed derivation of Eq.(10) of the main text, (V) High temperature regime for the case the thermal effect of the SC island is mainly from the external environment, and (VI) Discussion about $2e$ conductance peak.

I. EFFECTIVE LOW-ENERGY IMPURITY HAMILTONIAN AND CONDUCTANCE PEAK POSITION

In this section, we discuss the effective low-energy impurity Hamiltonian after neglecting the lead-impurity tunnelings λ_L and λ_R. Of this situation, the Hamiltonian of the island becomes

$$U_c = E_c(N - n_g)^2 + \nu\gamma_1\gamma_2, \tag{1}$$

where n_g indicates the energetically most-preferred occupation number in the dot, and γ_1, γ_2 refer to two quasi-MZMs (or two coupled regular MZMs) next to the left lead. For a standard Fu-teleportation, the coupling between two non-local MZMs modifies the peak position (represented by the value of n_g) where two impurity states become energy-degenerate. Indeed, in Fu-teleportation, energies of these two impurity states are both ν and n_g-dependent. By contrast, in our system the peak position is instead ν-independent.

To see this is indeed the case, we consider a small detuning δn from the half-filling (i.e., $n_g = 2n_0 + 1/2 + \delta n$), with which the impurity Hamiltonian can be presented in the matrix form

$$H_{\text{impurity}} = \begin{pmatrix} E_c\delta n & 0 & 0 & -i\nu \\ 0 & -E_c\delta n & i\nu & 0 \\ 0 & -i\nu & -E_c\delta n & 0 \\ i\nu & 0 & 0 & E_c\delta n \end{pmatrix}, \tag{2}$$

where matrix indices respectively represents impurity states $\{|00\rangle, |10\rangle, |01\rangle, |11\rangle\}$. After the exact diagonalization of H_{impurity}, we figure out its four eigenstates with their corresponding energies

$$\begin{aligned}
|\psi_1\rangle = |o_1\rangle &= \frac{1}{\sqrt{2}}\left(i|10\rangle + |01\rangle\right), & \epsilon_{o1} &= \nu - E_c\delta n, \\
|\psi_2\rangle = |e_2\rangle &= \frac{1}{\sqrt{2}}\left(-i|00\rangle + |11\rangle\right), & \epsilon_{e2} &= \nu + E_c\delta n, \\
|\phi_1\rangle = |o_2\rangle &= \frac{1}{\sqrt{2}}\left(-i|10\rangle + |01\rangle\right), & \epsilon_{o2} &= -\nu - E_c\delta n, \\
|\phi_2\rangle = |e_1\rangle &= \frac{1}{\sqrt{2}}\left(i|00\rangle + |11\rangle\right), & \epsilon_{e1} &= -\nu + E_c\delta n,
\end{aligned} \tag{3}$$

where e and o respectively label impurity states with even and odd parities. Assuming that $\nu > 0$, states $|\phi_1\rangle$ and $|\phi_2\rangle$ respectively have lower energies in comparison to that of $|\psi_1\rangle$ and $|\psi_2\rangle$. Clearly, when $\nu \neq 0$, degenerate states are possible only when $\delta n = 0$, i.e., when $n_g = 2n_0 + 1/2$ is indeed tuned to the half-filling. This criteria is the same of degeneracy for both low-energy and high-energy regimes discussed in the main text. Since degeneracy provides extra options in the tunneling of particles, we anticipate the occurrence of the 1e tunneling peak of our model at half filling, independent of the inter-MZM coupling constant ν. Briefly, this irrelevance of ν to the peak position grounds in the fact that γ_1 and γ_2 of our model are local in space. By contrast, two coupling MZMs of a common Fu-teleportation are responsible for the non-local transport.

II. EFFECTIVE LOW-ENERGY TUNNELING HAMILTONIAN

After obtaining eigenstates and their corresponding energies of the island, we discuss the influence of tunneling amplitudes. Here we assume that $\delta n = 0$, and focus only on the tunneling at the peak position. We check the effect of lead-island tunneling

operators on the island states, leading to

$$\gamma_1 e^{-i\varphi/2}|\psi_2\rangle = |\phi_1\rangle, \quad \gamma_1 e^{-i\varphi/2}|\phi_2\rangle = |\psi_1\rangle,$$
$$\gamma_4 e^{-i\varphi/2}|\phi_1\rangle = |\phi_2\rangle, \quad \gamma_4 e^{-i\varphi/2}|\psi_1\rangle = |\psi_2\rangle. \tag{4}$$

Supplementary Eq. (4) shows us the effect of $\gamma_1 e^{-i\varphi/2}$ and $\gamma_4 e^{-i\varphi/2}$: $\gamma_4 e^{-i\varphi/2}$ changes the parity state while conserving the system energy; while $\gamma_1 e^{-i\varphi/2}$ changes the impurity state between the low-energy states ($|\phi_1\rangle$ and $|\phi_2\rangle$) and the high-energy ones ($|\psi_1\rangle$ and $|\psi_2\rangle$).

Supplementary Eq. (4) provides an alternative way in the understanding of the effective spin operators in Eq. (3) of the main text: here the spin operator S_z models the change of energy (i.e., different S_y eigenvalues) induced by the impurity-left lead coupling. Following Supplementary Eq. (4), we understand the zero-conductance at zero energies as the lack of energy to visit the high-energy states ($|\psi_1\rangle$ and $|\psi_2\rangle$). Indeed, the operator $c^\dagger_{kL}\gamma_1 e^{-i\varphi/2}$ is forbidden at zero energies. In contrast, $c^\dagger_{kR}\gamma_4 e^{-i\varphi/2}$ is allowed, as it does not change the system energy. However, it alone can not produce a persistent current. Instead, a persistent current requires the higher order tunneling operator introduced in the main text that connects the island and the left lead. At zero energies, these higher-order processes have zero amplitudes following Eq. (4) of the main text.

As another key feature, in Eq. (3) of the main text we model the energy change induced by the impurity MZM γ_1 via the definition of the impurity operator $f_2 = |00\rangle\langle 11| - |10\rangle\langle 01| = -d_1 d_2 - d_1^\dagger d_2$. This operator is mappable to spin operators, as it satisfies the commutators: (i) $f_2^2 = (f_2^\dagger)^2 = 0 = S_-^2 = S_+^2$; (ii) $[f_2^\dagger f_2 - 1/2, f_2] = -f_2$ and $[f_2^\dagger f_2 - 1/2, f_2^\dagger] = f_2^\dagger$. These commutation relations perfectly agree with $[S_z, S_+] = S_+$ and $[S_z, S_-] = -S_-$ of spin operators. After substituting the impurity operator f_2 by the corresponding spin operators, we have arrived at Eq. (3) of the main text.

III. CALCULATION OF THE CURRENT

In this section, we provide details on the derivation of the zero-temperature current under an applied bias. As the starter, in the main text we have shown that the bare tunneling at the left side, i.e., $-2\lambda_L \sum_k c^\dagger_{kL} S_z f_1$ is energetically forbidden, as it connects two states with different energies. However, it can be used to construct tunnelings through virtual states. Indeed, with the Schrieffer-Wolff transformation [1], one can construct the higher-order tunneling Hamiltonian

$$\begin{aligned}\mathcal{O}_A &\sum_{p,q,k} \lambda_L c^\dagger_{pL} S_z f_1 \frac{1}{\nu - \epsilon_p} \lambda_R f_1^\dagger c_{kR} \frac{1}{\nu + \epsilon_q} \lambda_L c^\dagger_{qL} S_z f_1 \\ &\approx \sum_{\epsilon_p, \epsilon_q, k} \frac{2(\epsilon_p - \epsilon_q)}{\nu^3} \lambda_L^2 \lambda_R c^\dagger_{pL} c^\dagger_{qL} c_{kR} f_1,\end{aligned} \tag{5}$$

where in the second line we have expand to the leading order of lead-state energies ϵ_p and ϵ_q. For non-equilibrium or finite-temperature situations, $\epsilon_p - \epsilon_q$ respectively has the order of bias and temperature. It thus adds to the energy power-laws from RG analysis.

We visit the current through the superconducting island via the Greens function technique. In this work, since the only non-quadratic $\mathcal{O}_A$ is RG-irrelevant operator, we treat it perturbatively, while solving the rest of the Hamiltonian exactly. By doing so, the current operator becomes

$$\begin{aligned}\hat{I} &= -\partial_t [f_1^\dagger f_1 + \sum_k c^\dagger_{kR} c_{kR}] = -[H_{\mathrm{T}}, f_1^\dagger f_1 + \sum_{k'} c^\dagger_{k'R} c_{k'R}] \\ &= 2i \sum_{p>q,k} (t_{p,q} c^\dagger_{pL} c^\dagger_{qL} c_{kR} f_1 - h.c.) \equiv 2i(L - L^\dagger),\end{aligned} \tag{6}$$

where the minus sign is added to define the $R \to L$ current as positive, $t_{p,q} = 2(\epsilon_p - \epsilon_q)\lambda_L^2 \lambda_R/\nu^3$, and $L = \sum_{p>q} t_{p,q} c^\dagger_{pL} c^\dagger_{qL} c_{kR} f_1$.

We calculate the current at zero temperature T, under a bias V that is applied to the right lead. As a famous trick (see e.g., Refs. [2, 3]), one can deal with this bias with the transformation $c^\dagger_{kR} \to c^\dagger_{kR} \exp(ieVt)$, after which the calculation can be delta with as if the system was in equilibrium.

With this trick, to the leading order of t_L, current can be calculated as

$$I = 2\int_{-\infty}^{\infty} dt e^{ieVt} \Big\langle [L^\dagger(t), L(0)] \Big\rangle = 2\int_{-\infty}^{\infty} dt e^{iVt} \left\{ \Big\langle L^\dagger(t) L(0) \Big\rangle + \Big\langle L(0) L^\dagger(t) \Big\rangle \right\}. \tag{7}$$

The first part of this calculation equals

$$
\begin{aligned}
&\int_{-\infty}^{\infty} dt e^{ieVt} \sum_{p>q,k} \sum_{p'>q',k'} t_{p,q} t_{p',q'} \langle c_{qL}(t) c_{pL}(t) c^\dagger_{p'L} c^\dagger_{q'L} \rangle \langle f_1^\dagger(t) c^\dagger_{kR}(t) c_{k'R}(0) f_1(0) \rangle \\
=&\sum_{p>q} \sum_{k,k'} \int_{-\infty}^{\infty} dt e^{i(eV-\epsilon_p-\epsilon_q)t} t_{p,q} t_{p',q'} [1-n_F(\epsilon_p)][1-n_F(\epsilon_q)] \\
&\times \left[G^<(f_1^\dagger, c_{k'R}, -t) G^<(c^\dagger_{kR}, f_1, -t) - G^<(f_1^\dagger, f_1, -t) G^<(c^\dagger_{kR}, c_{k'R}, -t) \right] \\
=&\sum_{p>q} \sum_{k,k'} \int_{-\infty}^{\infty} d\omega' \frac{4\lambda_L^4 \lambda_R^2}{\nu^6} (\epsilon_p-\epsilon_q)^2 [1-n_F(\epsilon_p)][1-n_F(\epsilon_q)] \\
&\times \left[\tilde{G}^<(f_1^\dagger, c_{k'R}, \omega') \tilde{G}^<(c^\dagger_{kR}, f_1, -eV+\epsilon_p+\epsilon_q-\omega') - \tilde{G}^<(f_1^\dagger, f_1, \omega') \tilde{G}^<(c^\dagger_{kR}, c_{k'R}, -eV+\epsilon_p+\epsilon_q-\omega') \right],
\end{aligned} \tag{8}
$$

where n_F refers to the fermi distribution function. At zero temperature $T=0$, $n_F(\epsilon)=\Theta(-\epsilon)$ equals the step function.

Lesser Greens functions of Supplementary Eq. (8) can be obtained via standard method [4], with the result

$$
\begin{aligned}
&\sum_k \tilde{G}^<(f_1^\dagger, c_{kR}, \omega) = \sum_k \tilde{G}^<(c^\dagger_{kR}, f_1, \omega) = 2\pi i \rho \frac{\lambda_R \omega}{\omega^2+\Gamma_R^2} n_F(\omega) \\
&\tilde{G}^<(f_1^\dagger, f_1, \omega) = \frac{2i\Gamma_R}{\omega^2+\Gamma_R^2} n_F(\omega), \quad \sum_{k,k'} \tilde{G}^<(c^\dagger_{kR}, c_{k'R}, \omega) = 2\pi i \rho \frac{\omega^2}{\omega^2+\Gamma_R^2} n_F(\omega).
\end{aligned} \tag{9}
$$

Notice that the lead lesser Greens function contains an extra power of energy $\sim \omega^2$. This factor reflects the hybridization of the impurity f_1 by the right lead when $\Gamma_R \gg \omega \sim eV$.

With Supplementary Eq. (9), the target integral Supplementary Eq. (8) becomes

$$
\sum_{p>q} \frac{16\lambda_L^4 \Gamma_R^2}{\nu^6} (\epsilon_p-\epsilon_q)^2 \left[\frac{\arctan(\delta\omega)}{\Gamma_R} - \frac{1}{\delta\omega} \ln\left(\frac{\delta\omega^2+\Gamma_R^2}{\Gamma_R^2} \right) \right] \mathcal{S}_{p,q}. \tag{10}
$$

where $\delta\omega = eV - \epsilon_p - \epsilon_q$. Here the summation over p, q is taken in the area

$$
\mathcal{S}_{p,q} = \{\, p,\ q \mid \epsilon_p > 0, \epsilon_q > 0, \epsilon_p + \epsilon_q < eV \ \text{ and } \ \epsilon_p > \epsilon_q \,\}, \tag{11}
$$

which is a triangle in the (ϵ_p, ϵ_q) space. The full expression of the conductance becomes

$$
G = \frac{e^2}{h} \frac{32\Gamma_L^2 \Gamma_R^4}{3\pi^2 \nu^6} \left\{ -8\chi^2 + \chi\left[9+\chi^2\right] \arctan(\chi) + \left(-1+3\chi^2\right) \ln\left(1+\chi^2\right) + \frac{3}{2}\chi^2 \mathrm{Li}_2\left(-\chi^2\right) \right\}, \tag{12}
$$

where the energy ratio $\chi \equiv eV/\Gamma_R$, and Li_n refers to the polylogarithm function. In two limiting cases, the conductance approximately becomes

$$
\begin{aligned}
G &\approx \frac{e^2}{h} \frac{4\Gamma_L^2}{45\pi^2 \nu^6 \Gamma_R^2} (eV)^6, \qquad \text{when } eV \ll \Gamma_R, \\
G &\approx \frac{e^2}{h} \frac{16\Gamma_L^2 \Gamma_R}{3\pi \nu^6} (eV)^3, \qquad \text{when } eV \gg \Gamma_R.
\end{aligned} \tag{13}
$$

IV. 1E CONDUCTANCE OF THE CLEAN CASE IN $\nu \ll T \ll E_c$

The occupation probability of electrons at different energy levels can be described by rate equations

$$
\begin{aligned}
\dot{P}_\alpha &= -\sum_\beta \Gamma_{\alpha\to\beta} P_\alpha + \sum_\beta \Gamma_{\beta\to\alpha} P_\beta, \\
\dot{P}_\beta &= -\sum_\alpha \Gamma_{\beta\to\alpha} P_\beta + \sum_\alpha \Gamma_{\alpha\to\beta} P_\alpha,
\end{aligned} \tag{14}
$$

where $\dot{P}_\alpha$ and $\dot{P}_\beta$ are the occupation probability of even $\alpha = |e_1\rangle, |e_2\rangle$ and odd $\beta = |o_1\rangle, |o_2\rangle$ parity state, respectively, and $\Gamma_{i\to f} = \Gamma^L_{i\to f} + \Gamma^R_{i\to f} = \sum_j \Gamma^j_{i\to f}$ represents the transition probability between different impurity states $|i\rangle$ and $|f\rangle$. Assuming that the occupation probability of the single particle state in the lead follows the Fermi-Dirac distribution $f(\omega) = 1/(1+e^{\omega/kT})$, the following expressions of $\Gamma^j_{i\to f}$ are obtained from the Fermi golden rule:

$$\begin{aligned}\Gamma^j_{\alpha\to\beta} =& \frac{2\Gamma_j}{\hbar}\int d\xi_p\, \delta\left(E_\alpha - E_\beta + \xi_p\right) f\left(\xi_p - \mu_j\right),\\ \Gamma^j_{\beta\to\alpha} =& \frac{2\Gamma_j}{\hbar}\int d\xi_p\, \delta\left(E_\beta - E_\alpha - \xi_p\right)\left[1 - f\left(\xi_p - \mu_j\right)\right],\end{aligned} \tag{15}$$

where $\mu_L = eV, \mu_R = 0, \Gamma_j = \pi\rho|\lambda_j^2|$ describes transition amplitude, ρ is the density of states, $E_\beta - E_\alpha$ is the energy difference between odd β and even α parity states, and ξ_p is the electron energy in the leads.

In the steady state, rate equations become $\dot{P}_\alpha = 0, \dot{P}_\beta = 0$. Combining the normalization conditions $\sum_\alpha P_\alpha + \sum_\beta P_\beta = 1$, we can work out the occupation probabilities of the four impurity states $P_{|e_1\rangle}$, $P_{|e_2\rangle}$, $P_{|o_1\rangle}$ and $P_{|o_1\rangle}$. Then the steady current can be easily calculated, the expression of the current is

$$I = e\sum_{\alpha,\beta} P_\alpha \Gamma^L_{\alpha\to\beta} - e\sum_{\alpha,\beta} P_\beta \Gamma^L_{\beta\to\alpha} \tag{16}$$

From Supplementary Eq. (4), only two specific transitions $|e_1\rangle \to |o_1\rangle$ and $|e_2\rangle \to |o_2\rangle$ are allowed in the tunneling between γ_1 and the left lead, so we have

$$\begin{aligned}\sum_{\alpha,\beta} P_\alpha \Gamma^L_{\alpha\to\beta} =& P_{e_1}\Gamma^L_{e_1\to o_1} + P_{e_2}\Gamma^L_{e_2\to o_2},\\ \sum_{\alpha,\beta} P_\beta \Gamma^L_{\beta\to\alpha} =& P_{o_1}\Gamma^L_{o_1\to e_1} + P_{o_2}\Gamma^L_{o_2\to e_2}.\end{aligned} \tag{17}$$

At zero bias, using the formula of the differential conductance $G = \frac{\partial I}{\partial V}\big|_{V\to 0}$, the tunneling conductance reads as

$$G = \frac{e^2}{2T\hbar}\frac{\Gamma_L\Gamma_R}{\Gamma_L+\Gamma_R}\text{sech}(\frac{\nu}{T})^2\text{sech}[\frac{E_c(1-2\delta_g)}{2T}]^2. \tag{18}$$

For Majorana case with level spacing 2ν, the analytical method is the same, while the impurity states in the island reduce to two eigenstates (even stste $|o\rangle$ and odd state $|o\rangle$). In the large temperautre limit $\nu/T \to 0$, we can easily obtain the expression of conductance for the standard Fu-teleportation [5]

$$G_{Maj} = \frac{e^2}{2T\hbar}\frac{\Gamma_L\Gamma_R}{\Gamma_L+\Gamma_R}\text{sech}[\frac{2\nu + E_c(1-2\delta_g)}{2T}]^2. \tag{19}$$

V. 1E CONDUCTANCE OF THE DIRTY CASE IN $\nu \ll T \ll E_c$

Actually, the above calculation of the current is self-consistent and corresponds to a "clean" transport process, where the energy levels in the island are discrete and the island is only coupled with two leads. However, if the nanowire is also affected by disorder or the external environment, the self-consistency will be broken. In this case, the occupation of the energy levels is sophisticated and the entire island is more like in thermal equilibrium. We call this situation "dirty" transport. In this case, the occupation probabilities of different electronic states are proportional to the Fermi distribution function

$$\begin{aligned}P_{e_1} = P_0\frac{1}{e^{-\nu/kT}+1}, \quad P_{e_2} = P_0\frac{1}{e^{\nu/kT}+1},\\ P_{o_1} = P_1\frac{1}{e^{\nu/kT}+1}, \quad P_{o_2} = P_1\frac{1}{e^{-\nu/kT}+1},\end{aligned} \tag{20}$$

where $P_{0/1}$ represents the probability to find the nanowire in the even/odd parity electronic state. In the steady state, the current satisfy

$$I = I_L = -I_R, \tag{21}$$

where I_L is expressed in Supplementary Eq. (16), similarly I_R is obtained by changing the superscript of the transition probability in I_L from L to R

$$I_R = e\sum_{\alpha,\beta} P_\alpha \Gamma^R_{\alpha\to\beta} - e\sum_{\alpha,\beta} P_\beta \Gamma^R_{\beta\to\alpha}. \tag{22}$$

From Supplementary Eq. (4), we have

$$\begin{aligned}\sum_{\alpha,\beta} P_\alpha \Gamma^R_{\alpha\to\beta} &= P_{e_1}\Gamma^R_{e_1\to o_2} + P_{e_2}\Gamma^R_{e_2\to o_1},\\ \sum_{\alpha,\beta} P_\beta \Gamma^R_{\beta\to\alpha} &= P_{o_1}\Gamma^R_{o_1\to e_2} + P_{o_2}\Gamma^L_{o_2\to e_1}.\end{aligned} \tag{23}$$

It is worth noting that although the expression of I_R seems to be obtained only by changing the superscript of the transition probability in I_L from L to R, however, because of the asymmetry, the transition of the impurity states has become different (i.e., left is $|e_1\rangle \to |o_1\rangle$ while right is $|e_1\rangle \to |o_2\rangle$).

Similarly, substituting Supplementary Eq. (16) and Supplementary Eq. (22) into Supplementary Eq. (21), then combining normalization equation $P_0 + P_1 = 1$, one can figure out the differential conductance at zero-bias voltage

$$\begin{aligned}G =& \frac{e^2\Gamma_L\Gamma_R}{T\hbar}\\ &\times \frac{\Gamma_L + 2\Gamma_R - 2\Gamma_R\cosh\left(\frac{\nu}{T}\right) + \Gamma_R\cosh\left(\frac{2\nu}{T}\right) + (\Gamma_L+\Gamma_R)\left[-1+2\cosh\left(\frac{\nu}{T}\right)\right]\cosh\left[\frac{E_c(1-2\delta_g)}{T}\right]}{\left(-\Gamma_L + 2\Gamma_L\cosh\left(\frac{\nu}{T}\right) + \Gamma_R\cosh\left(\frac{2\nu}{T}\right) + (\Gamma_L+\Gamma_R)\cosh\left[\frac{E_c(1-2\delta_g)}{T}\right]\right)^2}.\end{aligned} \tag{24}$$

At $\delta_g = 1/2$, the peak conductance becomes

$$G_{\text{peak}} = \frac{e^2}{2T\hbar}\frac{\Gamma_L\Gamma_R\,\text{sech}\left(\frac{\nu}{T}\right)}{\Gamma_L + \Gamma_R\cosh\left(\frac{\nu}{T}\right)}, \tag{25}$$

VI. $2e$ CONDUCTANCE PEAK

As has been shown in the main text, a $1e$ conductance peak occurs only in an island with half filling (where n_g is a half integer). Instead, $2e$ conductance dominates when n_g is an odd integer. In this section, we evaluate the $2e$ peak conductance with the rate equation method. For later convenience, we define $|0\rangle, |1\rangle, |2\rangle$ as the states with N, $N+1$, and $N+2$ particles in the island, respectively. For brevity, we also define the notation $\sum_{\alpha,\beta} P_\alpha\Gamma_{\alpha\to\beta} \to P_0\Gamma_{0\to1}$. With these notations, the rate equations become

$$\begin{aligned}\dot{P}_0 &= -\left(\Gamma_{0\to1} + \Gamma_{0\to2}\right)P_0 + P_1\Gamma_{1\to0} + P_2\Gamma_{2\to0},\\ \dot{P}_1 &= -\left(\Gamma_{1\to0} + \Gamma_{1\to2}\right)P_1 + P_0\Gamma_{0\to1} + P_2\Gamma_{2\to1},\\ \dot{P}_2 &= -\left(\Gamma_{2\to0} + \Gamma_{2\to1}\right)P_2 + P_0\Gamma_{0\to2} + P_1\Gamma_{1\to2},\end{aligned} \tag{26}$$

where $\Gamma_{0\to1,1\to0} = \sum_j \Gamma^j_{0\to1,1\to0}$ is defined in Supplementary Eq. (15), $\Gamma^j_{1\to2,2\to1}$ can be obtained by sending $E_1 - E_0 \to E_2 - E_1$. Meanwhile, following Ref. [5], we obtain the $2e$ transition rate

$$\begin{aligned}\Gamma^j_{0\to2} &= \frac{2A_j}{\hbar}\frac{E_2 - E_0 - 2\mu_j}{e^{E_2-E_0-2\mu_j} - 1},\\ \Gamma^j_{2\to0} &= \frac{2A_j}{\hbar}\frac{E_0 - E_2 + 2\mu_j}{e^{E_0-E_2+2\mu_j} - 1},\end{aligned} \tag{27}$$

where $\mu_L = eV, \mu_R = 0$. $A_j \sim \Gamma_j^2/\Delta_{sc}^2$ is a dimensionless parameter describing the strength of Andreev reflection, Δ_{sc} is the superconducting gap, and $E_2 - E_0$ is the energy difference between states $|0\rangle$ and $|2\rangle$. With the rate equation solutions, the total current becomes

$$I_{\text{total}} = I_{1e} + I_{2e}, \tag{28}$$

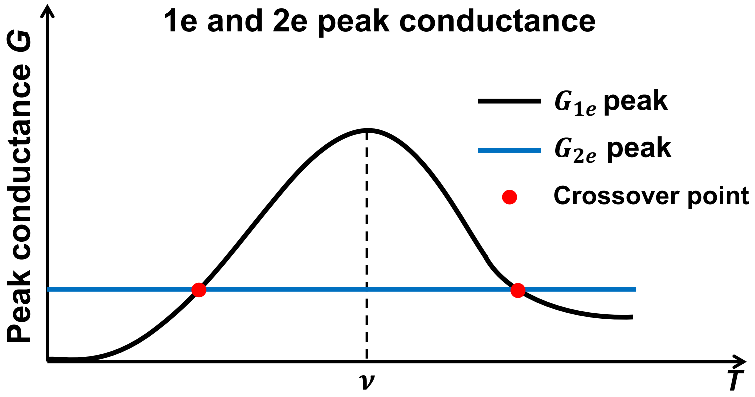

Supplementary Figure 1. The $1e$ and $2e$ conductance peak values as functions of ν. Although we plot the conductance values in a single plot, the $1e$ and $2e$ conductance peaks can not appear simultaneously. Indeed, their appearance requires distinct values of n_g.

where the first contribution of the single electron tunneling is

$$I_{1e} = e\left(P_0\Gamma^L_{0\to1} + P_1\Gamma^L_{1\to2}\right) - e\left(P_1\Gamma^L_{1\to0} + P_2\Gamma^L_{2\to1}\right), \tag{29}$$

and the second contribution of Andreev reflection is

$$I_{2e} = 2e\left(P_0\Gamma^L_{0\to2} - P_2\Gamma^L_{2\to0}\right). \tag{30}$$

In agreement with our analysis above, when $\delta_g = 1$, single-electron transport is exponentially suppressed by the factor $\exp(E_c/kT)$, leading to $\Gamma_{0\to1} = \Gamma_{2\to1} \approx 0$. Taking this condition into Supplementary Eq. (26), we get $P_1 \approx 0$. In steady state, by solving rate equations and normalized condition, we obtain the 2e conductance

$$G_{2e} = \frac{4e^2}{\hbar}\frac{A_L A_R}{A_L + A_R}\frac{4E_c\left(1-\delta_g\right)/T}{\sinh\left(4E_c\left(1-\delta_g\right)/T\right)}, \tag{31}$$

After tuning the voltage such that $\delta_g = 1$, Andreev reflection [5] contribution dominates, where $2e$ conductance reaches the maximum value

$$G^{peak}_{2e} = \frac{4e^2}{\hbar}\frac{A_L A_R}{A_L + A_R}. \tag{32}$$

We can compare the $1e$ and $2e$ conductance values in the regime with $\nu \ll T \ll E_c$. Briefly, as shown in Supplementary Fig. 1, the $2e$ conductance peak is higher than the $1e$ conductance peak when ν is either large enough or small enough. Importantly, although we plot the $1e$ and $2e$ conductance peak values in the same plot, we emphasize that $1e$ and $2e$ conductance peaks have distinct prerequisites (to occur), and thus can not occur simultaneously in real experiments.

Without loss of generality, we assume that the point contacts at both terminals are symmetrical $\Gamma_L = \Gamma_R = \Gamma/2$. In high-energy regime, $G^{peak}_{1e}/G^{peak}_{2e} \sim \Delta^2_{sc}/(\Gamma T)$, and one may find there will be a crossover point around $T = \Delta^2_{sc}/\Gamma$. In low-energy regime, combining Eq. (7) and Eq. (8) of the main text, another crossover point should appear around $T = \Gamma$. Therefore, we predict that there will be two crossover points on both sides of $T \sim \nu$, as shown in Supplementary Fig. 1.

In real experiments, this unique double-crossover structure can also help us to indirectly detect the anomalous temperature dependence of Coulomb blockade conductance. Indeed, generically the $1e$ and $2e$ conductance peak values cross at a single point.

[1] A. C. Hewson, *The Kondo problem to heavy fermions*, Vol. 2 (Cambridge university press, 1997).
[2] C. L. Kane and M. P. A. Fisher, Phys. Rev. B **46**, 7268 (1992).
[3] C. de C. Chamon, D. E. Freed, and X. G. Wen, Phys. Rev. B **53**, 4033 (1996).
[4] H. Bruus and K. Flensberg, *Many-body quantum theory in condensed matter physics - an introduction* (Oxford University Press, United States, 2004).
[5] B. van Heck, R. M. Lutchyn, and L. I. Glazman, Phys. Rev. B **93**, 235431 (2016).
